# NQO1 regulates expression and alternative splicing of apoptotic genes associated with Alzheimer's disease in PC12 cells

**DOI:** 10.1002/brb3.2917

**Published:** 2023-03-31

**Authors:** Yingshi Du, Gejing Liu, Dong Chen, Jinggang Yang, Jing Wang, Yue Sun, Qian Zhang, Yongming Liu

**Affiliations:** ^1^ Section 1, Department of Geriatrics The First Hospital of Lanzhou University Lanzhou China; ^2^ Center for Genome Analysis ABLife Inc. Wuhan China

**Keywords:** alternative splicing (AS), Alzheimer's disease (AD), apoptosis, quinone oxidoreductase 1 (NQO1), RNA sequencing (RNA‐seq), transcriptional regulation

## Abstract

**Purpose:**

Alzheimer's disease (AD) is a neurodegenerative disorder characterized by progressive memory loss and cognitive dysfunction. Quinone oxidoreductase 1 (NQO1) is an antioxidant enzyme that plays an important role in controlling cellular redox state, whose expression is altered in the brain tissues of AD patients. In addition to its traditional antioxidant effects, NQO1 also acts as a multifunctional RNA‐binding protein involved in posttranscriptional regulation. Whether the RNA‐binding activity of NQO1 influences AD pathology has not been investigated yet.

**Methods:**

The RNA‐binding functions of NQO1 in rat pheochromocytoma (PC12) cells were investigated using siRNA knockdown followed by total RNA sequencing. Reverse transcription quantitative polymerase chain reaction was performed to explore the impact of NQO1 on the transcription and alternative splicing of apoptotic genes.

**Results:**

NQO1 knockdown led to a significant increase in cellular apoptosis. Genes involved in certain apoptosis pathways, such as positive regulation of apoptotic processes and mitogen‐activated protein kinase signaling, were under global transcriptional and alternative splicing regulation. NQO1 regulated the transcription of apoptotic genes *Cryab*, *Lgmn*, *Ngf*, *Apoe*, *Brd7*, and *Stat3*, as well as the alternative splicing of apoptotic genes *BIN1*, *Picalm*, and *Fyn*.

**Conclusion:**

Our findings suggest that NQO1 participates in the pathology of AD by regulating the expression and alternative splicing of the genes involved in apoptosis. These results extend our understanding of NQO1 in apoptotic pathways at the posttranscriptional level in AD.

## Graphical abstract

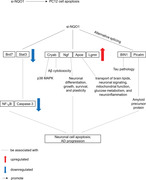

## 1 INTRODUCTION

Alzheimer's disease (AD), characterized by progressive cognitive dysfunction, is regarded as the most common cause of dementia in the elderly. According to data released by Alzheimer's Disease International (ADI), the global prevalence of dementia was once estimated to be as high as 55 million in 2020, and the prevalence is predicted to be 139 million by 2050, which poses an ever‐greater public health burden (Rimmer, 2006).

AD etiology and pathogenesis are not fully understood. Studies have shown that amyloid beta (Aβ) peptide plays a critical role in the initiation of AD pathology (Hardy & Higgins, 1992; Selkoe & Hardy, 2016). Aβ oligomers also induce abnormal tau hyperphosphorylation, which promotes the formation of neurofibrillary tangles (Fan et al., 2019). Therefore, the search for AD therapies has mainly focused on these two main hallmarks of the disease. Unfortunately, attempts to target Aβ‐mediated pathology have been largely unsuccessful (Feldman et al., 2014; Karran et al., 2011), and tau‐targeting strategies are still being explored (Congdon & Sigurdsson, 2018). It seems likely that numerous complex mechanisms lead to the deposition of the two major proteins in this disease.

Another strategy to treating AD may lie in preventing oxidative stress that plays a key role in the disease (Butterfield & Halliwell, 2019; Huang et al., 2016; Tramutola et al., 2017; Yan et al., 2013). The brain contains abundant easily oxidizable lipids and it consumes large amounts of oxygen, rendering the organ quite vulnerable to oxidative stress (Halliwell, 2006). The presence of Aβ plaques has been linked to oxidative stress since mitochondrial accumulation of Aβ reduces oxygen consumption and decreases electron transport chain activity (Manczak et al., 2006). At the same time, oxidative stress has been shown to stimulate the production of Aβ (Nishida et al., 2006), and it may contribute to tau hyperphosphorylation and polymerization (Gamblin et al., 2000).

Quinone oxidoreductase 1 (NQO1) is a redox‐regulated flavoenzyme that plays a crucial role in controlling the cellular redox state (Dinkova‐Kostova & Talalay, 2010; Siegel et al., 2018). NQO1 catalyzes the two‐electron reduction of quinones and other related substrates, preventing their participation in redox cycling and subsequent generation of reactive oxygen species (Dinkova‐Kostova & Talalay, 2010; Ross & Siegel, 2004; Siegel et al., 2018). Since oxidative stress is associated with AD, it is not surprising that NQO1 is involved in AD pathogenesis (Chhetri et al., 2018; Raina et al., 1999; SantaCruz et al., 2004; Torres‐Lista et al., 2014; Tsvetkov et al., 2011). Studies of brain tissue from AD patients showed NQO1 upregulation specifically in glial cells, followed in later stages by upregulation in hippocampal neurons (Raina et al., 1999; SantaCruz et al., 2004). Studies in the triple‐transgenic mouse AD model showed NQO1 upregulation in the hippocampus and cortex in early stages of the disease, followed by a gradual decrease in expression (Torres‐Lista et al., 2014). Controversially, another study revealed that approximately half of AD patients may lack NQO1 expression entirely in the hippocampus, which has been attributed to a C609T polymorphism (Tsvetkov et al., 2011). Altogether, these studies suggest that the regulation of NQO1 is still unclear in AD progression.

In addition to its role as an antioxidant enzyme, NQO1 also acts as an RNA‐binding protein (RBP) (Di Francesco et al., 2016) that can regulate gene expression posttranscriptionally (Gerstberger et al., 2014). This led us to ask whether the RBP activity of NQO1 may be important in AD. To investigate this question, we knocked down the gene in rat pheochromocytoma (PC12) cells that are widely used in AD studies. Then, we performed high‐throughput RNA sequencing (RNA‐seq) to identify differentially expressed genes (DEGs) and alternative splicing events (ASEs) regulated by NQO1. Our results revealed obvious changes in the transcriptome profiles and splicing patterns of certain subsets of genes after NQO1 knockdown. The study might provide an important basis for further clarifying the role of NQO1 in AD progression.

## 2 MATERIALS AND METHODS

### 2.1 Cell culture and transfections

PC12 cells (CL‐0481, Procell Life Science & Technology Co., Ltd., Wuhan, Hubei, China) were cultured in RPMI‐1640 containing 10% fetal bovine serum (FBS, 164210, Procell, Wuhan, Hubei, China), 100 μg/mL streptomycin (SV30010, Hyclone), and 100 U/mL penicillin (Hyclone) at 37°C in an atmosphere of 5% carbon dioxide. Short interfering RNA (siRNA) targeting NQO1 and the control siRNA were transfected into cells using Lipofectamine 2000 (Invitrogen, Carlsbad, CA, USA) according to the manufacturer's manual. All siRNA duplexes were purchased from Gemma (Suzhou, China). The sequences were nontargeting control siRNA (siCtrl) (5′‐UUCUCCGAACGUGUCACGUTT‐3′ [sense]) and siRNA targeting NQO1 (siNQO1) (5′‐CUGACCUCUAUGCUAUGAATT‐3′ [sense]).

### 2.2 Assessment of NQO1 gene expression

After 48 h of transfection, cells were harvested and subjected to quantitative reverse transcription PCR (RT‐qPCR) analysis. Complementary DNA (cDNA) was synthesized using Prime Scrip RT Reagent kit (Takara, Dalian, China). RT‐qPCR was performed on the Bio‐Rad S1000 using Bestar SYBR Green RT‐PCR Master Mix (DBI Bioscience, Shanghai, China). Levels of target mRNAs were determined using the 2–ΔΔCT method and normalized to the levels of glyceraldehyde‐3‐phosphate dehydrogenase (GAPDH) mRNA (Livak & Schmittgen, 2001).

Intergroup differences in expression levels were assessed for significance using the paired Student's *t*‐test in GraphPad Prism software (version 7, San Diego, CA, USA).

### 2.3 Flow cytometry of cell apoptosis

PC12 cells were plated onto 24‐well culture plates (ca. 105 per well) and incubated for 24 h. Cells were double stained with Fluor647‐conjugated annexin V and propidium iodide (4A Biotech Co. Ltd., Beijing, China), and apoptosis was detected by flow cytometry (Cytoflex, Beckman, USA).

### 2.4 RNA extraction and sequencing

Cells were homogenized and total RNA was extracted using TRIzol (Ambion, Austin, TX, USA). RQ1 DNase (Promega, Fitchburg, WI, USA) was applied to remove DNA. The purified RNA was quantified by measuring the absorbance ratio at 260–280 nm using a Smartspec Plus system (BioRad, Hercules, CA, USA), and 1.5% agarose gel electrophoresis was performed to verify RNA integrity.

Total RNA (1 μg) from each sample was used for RNA‐seq library preparation with the KAPA Stranded mRNA‐Seq Kit for Illumina^®^ Platforms (#KK8544, Roche, Basel, Switzerland). Polyadenylated mRNAs were purified with VAHTS mRNA capture Beads (N401‐01, Vazyme, Nanjing, China), and fragmented mRNAs were converted into double‐stranded cDNA. Following end repair and A tailing, the DNAs were ligated to Diluted Roche Adaptor (KK8726). Ligation products corresponding to 300–500 base pairs were amplified, purified, quantified, and stored at −80°C before sequencing. The strand marked with 2′‐deoxyuridine 5′‐triphosphate (second cDNA strand) was not amplified, allowing strand‐specific sequencing. Libraries were prepared for high‐throughput sequencing according to the manufacturer's instructions, and 150 nt paired‐end sequencing was performed on an Illumina Novaseq 6000 system (ABlife Inc, Wuhan, China).

### 2.5 Cleaning and alignment of raw RNA‐seq data

Raw reads containing more than 2‐N bases were first discarded. Then, we used the FASTX‐Toolkit (Version 0.0.13) to trim adaptors and low‐quality bases from the raw sequencing reads. Reads shorter than 16 nucleotides were discarded. The remaining clean reads were aligned to the RGSC6 genome using TopHat2 (Kim et al., 2013) and allowing four mismatches. Uniquely mapped reads were used for gene read counting and calculation of the fragments per kilobase of transcript per million mapped reads (FPKM) (Trapnell et al., 2010).

### 2.6 DEG analysis

The R Bioconductor package edgeR (Robinson et al., 2010) was used to screen for DEGs differing between NQO1 knockdown cells and control PC12 cells. A false discovery rate (FDR) <0.05 and fold change >2 or <0.5 were set as the cutoff criteria for identifying DEGs.

### 2.7 Alternative splicing analysis

ASEs were identified and quantified using the ABLAS pipeline as described (Jin et al., 2017; Xia et al., 2017). The following 10 types of ASEs were detected based on the splice junction reads: intron retention (IntronR), exon skipping (ES), alternative 5′ splice site (A5SS), alternative 3′splice site (A3SS), mutually exclusive exons (MXE), mutually exclusive 5′UTRs (5pMXE), mutually exclusive 3′UTRs (3pMXE), cassette exon, A3SS&ES, and A5SS&ES.

To identify NQO1‐regulated ASEs, the ratio alteration of ASEs was assessed for significance using Student's *t*‐test. ASEs were significant at *p*‐value cutoff equivalent to an FDR cutoff of 5% and were considered to be regulated by NQO1.

### 2.8 RT‐qPCR validation of DEGs and ASEs

Some DEGs identified by RNA‐seq were validated using RT‐qPCR. Total RNA remaining from RNA‐seq library preparation was subjected to RT‐qPCR. The reverse transcription of RNA into cDNA was performed using M‐MLV reverse transcriptase (Vazyme). RT‐qPCR was performed on the StepOne RT‐PCR System (AT311‐03, Transgene, Beijing, China), using the SYBR Green PCR Reagents Kit (Low Rox plus, YEASEN, Shanghai, China) and sequences of primers in Table S1. The PCR conditions were as follows: denaturation at 95°C for 10 min, 40 cycles of denaturation at 95°C for 15 s, and then annealing and extension at 60°C for 1 min. For each sample, PCR amplifications were performed three times. Gene expression was normalized to that of GAPDH mRNA.

ASEs were similarly validated using RT‐qPCR and the primers in Table S1. To detect alternative isoforms, a boundary‐spanning primer for the sequence encompassing the junction of the constitutive exon and the alternative exon and an opposing primer in a constitutive exon were used. The boundary‐spanning primer of the alternative exon was designed according to the “model exon” to detect model splicing or according to “altered exon” to detect altered splicing.

### 2.9 Functional enrichment analysis

To predict the functional categories of DEGs, the KOBAS 2.0 server was used to identify gene ontology (GO) terms and the Kyoto Encyclopedia of Genes and Genomes (KEGG) pathways (Xie et al., 2011). Enrichment was determined based on the hypergeometric test and Benjamini–Hochberg FDR procedure.

## 3 RESULTS

### 3.1 NQO1 knockdown increases apoptosis of PC12 cells

**FIGURE 1:**
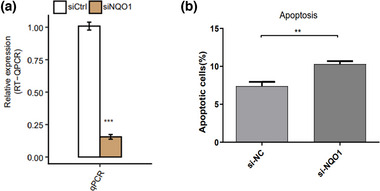
NQO1 knockdown enhances PC12 cell apoptosis. (a) NQO1 was knocked down using short interfering RNA targeting NQO1 (si‐NQO1) in PC12 cells. Control cells were transfected with a negative‐control siRNA. NQO1 expression was quantified using RT‐qPCR. (b) The apoptosis of NQO1 knockdown cells or control cells was determined by flow cytometric analysis. Data are shown as the mean ± standard deviation. Student's *t*‐test was used to analyze differences between two groups. **p < .01, ***p < .001.

To explore the role of NQO1 in AD, we knocked down NQO1 expression in PC12 cells through si‐NQO1 transfection and verified the reduced expression using RT‐qPCR (Figure 1a). Then we compared the percentage of apoptotic cells in si‐NQO1 group and si‐NC group. Knockdown of NQO1 led to a significant increase in cell apoptosis (Figure 1b).

### 3.2 NQO1 knockdown alters the transcriptome of PC12 cells

**FIGURE 2:**
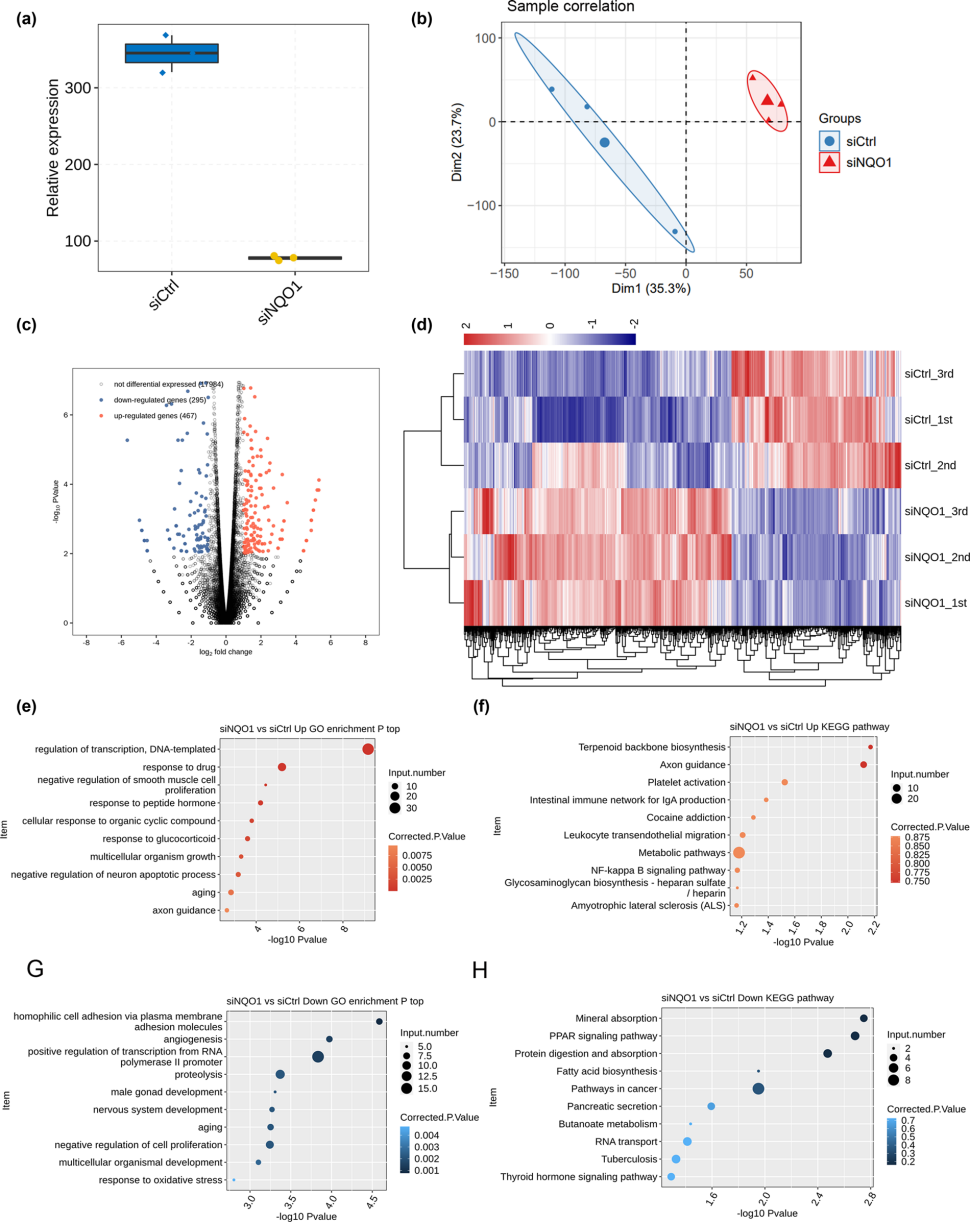
NQO1 regulates expression of genes involved in apoptosis. (a) NQO1 expression in NQO1 knockdown (siNQO1) group and control (siCtrl) group was quantified by RNA‐seq. (b) Transcriptional profiles of siNQO1 and siCtrl samples were separated by principal component analysis. (c) NQO1‐regulated genes were identified. Upregulated genes are labeled in red, and downregulated genes are labeled in blue. (d) Hierarchical clustering of differentially expressed genes (DEGs). Fragments per kilobase of transcript per million mapped reads (FPKM) values were log2‐transformed and then median‐centered for each gene. (E‐F) Upregulated DEGs were analyzed to reveal enrichment in (E) gene ontology (GO) biological processes and (F) Analysis of Kyoto Encyclopedia of Genes and Genomes (KEGG) pathways. Only the top 10 results are shown. (G, H) Downregulated DEGs were analyzed to reveal enrichment in (G) GO processes and (H) KEGG pathways. Error bars represent mean ± standard error of the mean (SEM). ***p < .001.

We compared the transcriptomes of PC12 cells transfected with si‐NQO1 or siCtrl using RNA‐seq. After removing sequence adaptors and low‐quality reads, an average of 85.4 million clean pair‐end reads per sample were obtained. These reads were mapped to the RGSC6 genome, resulting in an average of 76.1 million uniquely mapped read pairs per sample. The sequencing showed 15,421 expressed genes with FPKM > 0 and 10,138 genes with FPKM > 1 in at least one sample. The sequencing further confirmed NQO1 knockdown (Figure 2a). Principal component analysis (PCA) revealed that the first component clearly separated genome‐wide gene expression profiles of PC12‐siNQO1 cell samples from those of control cell samples, suggesting that the experimental replicates strongly correlated with one other, giving reliable results appropriate for subsequent analysis (Figure 2b).

Analysis with edgeR identified DEGs in PC12 cells transfected with siNQO1 and siCrt, revealing 467 upregulated genes and 295 downregulated genes. This suggests that NQO1 knockdown regulates expression of many genes in PC12 cells. A volcano plot was provided to show DEGs associated with NQO1 knockdown (Figure 2c). Heat maps of DEG expression patterns showed strong consistency within the biological replicates of PC12‐siNQO1 cell samples or control cell samples (Figure 2d).

To begin to examine the potential functions of these DEGs, GO enrichment analysis was performed. The top biological processes involving NQO1‐regulated genes are presented in Figure 2e,g. The genes upregulated in NQO1 knockdown cells were associated mainly with negative regulation of smooth muscle cell proliferation and neuron apoptotic processes, transcription regulation, DNA‐templated synthesis, axon guidance, and aging (Figure 2e). The genes downregulated in NQO1 knockdown cells were associated mainly with negative regulation of cell proliferation, nervous system development, response to oxidative stress, and other pathways related to AD (Figure 2g). Interestingly, the upregulated and downregulated DEGs were significantly enriched in apoptotic‐related processes, which ranked among the top 10 terms (Figure 2e). As shown by KEGG pathway analysis, the DEGs were enriched in axon guidance, nuclear factor‐kappa B signaling pathway, fatty acid biosynthesis, metabolic pathways, and cancer pathways (Figure 2f,h).

### 3.3 NQO1 regulates the expression of apoptosis‐related genes

**FIGURE 3:**
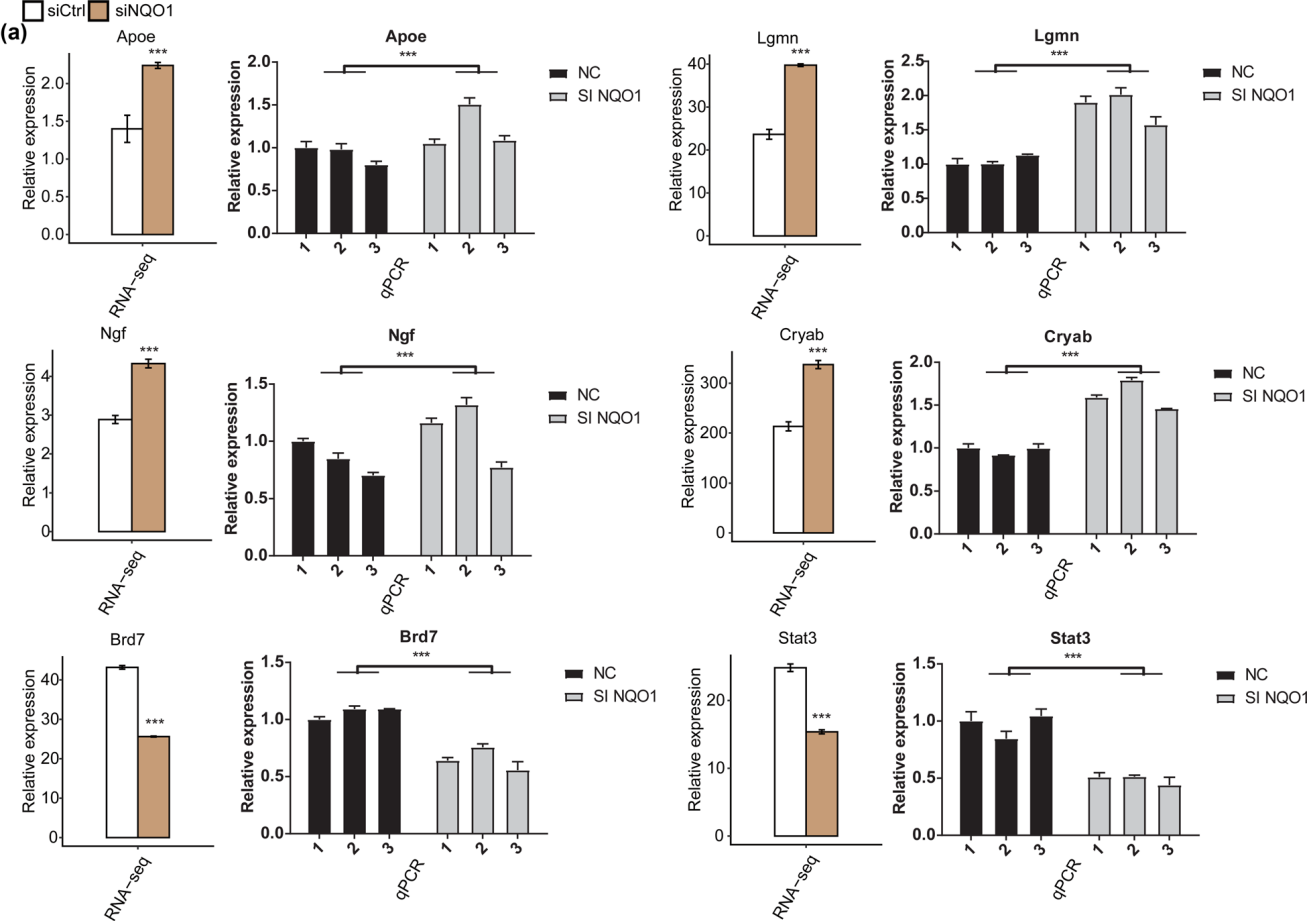
Validation by RT‐qPCR of certain NQO1‐regulated genes first identified by RNA sequencing. (a) Sequencing results are shown on the left, and RT‐qPCR results are presented on the right. Experimental data are shown as the mean ± standard deviation of at least three independent experiments. ***p < .001.

To verify the impact of NQO1 silencing on DEG expression, we performed RT‐qPCR to detect the expression of six DEGs (Cryab, Lgmn, Ngf, Apoe, Brd7, and Stat3) that have been reported to participate in apoptosis pathology. All these DEGs were associated with the GO molecular functional terms “negative regulation of neuron apoptotic processes,” “negative regulation of cell proliferation,” and “aging,” and the FPKM of all these genes was >1 in at least one RNA‐seq sample. The results of RT‐qPCR confirmed that Cryab, Lgmn, Ngf, and Apoe were upregulated in PC12 cells silencing NQO1, while Brd7 and Stat3 were downregulated by siNQO1 (Figure 3a).

### 3.4 NQO1 regulates the alternative splicing of genes implicated in apoptosis

The splice reads from PC12 cells transfected with siNQO1 or siCtrl were mapped to the reference genome, and 164,821 annotated exons were detected, accounting for 68.6% of all annotated reads. Then, 68,038 known and 240,124 novel splice junctions were detected using TopHat2. ASEs were identified from the splice junctions using ABLAS, which detected 1534 known and 38,038 novel ASEs.

**FIGURE 4:**
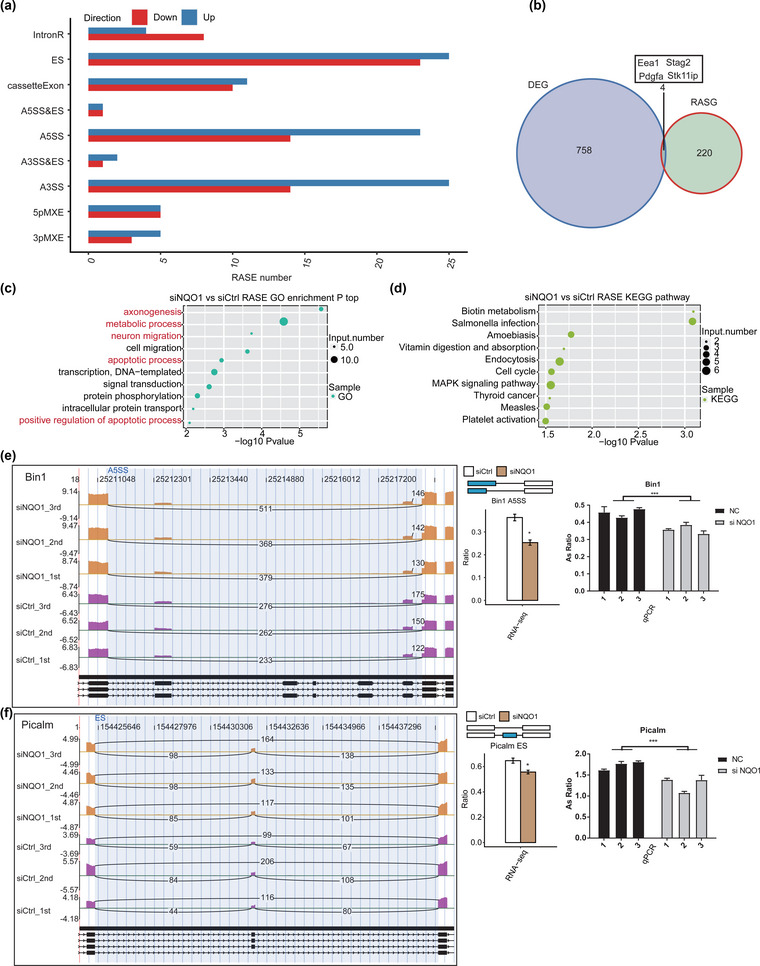
Identification and functional analysis of NQO1‐regulated alternative splicing events. (a) Frequency distribution of different types of NQO1‐regulated alternative splicing events. (b) Venn diagram showing the number of RASGs and DEGs as well as the name of four overlap genes. (c) The top 10 GO biological processes of RASGs. (d) The top 10 representative KEGG pathways of RASGs. (e, f) NQO1‐regulated cassette exon of BIN1 and Picalm. ASE changes in NQO1 knockdown cells and control cells were shown by Integrative Genomics Viewer (IGV)‐sashimi plots (left panel), and the transcripts for the gene are shown below. The schematic diagrams depict the structures of ASEs (right panel, top). The constitutive exon sequences are denoted with white boxes, intron sequences with horizontal line, and alternative exons with blue boxes. RNA‐seq quantification of ASEs is displayed at the bottom of the right panel. The altered ratio of ASEs in RNA‐seq was calculated according to the formula: alternative splice junction reads / (alternative splice junction reads + model splice junction reads), while the altered ratio of ASEs in RT‐qPCR was calculated using the formula: alternative splice transcripts level / model splice transcripts level. Student's *t*‐test was performed to compare differences between NQO1 knockdown cells and control cells. Error bars represent mean ± standard error of the mean (SEM). ***p < .001, **p < .01, *p < .05.

After applying stringent screening conditions (see Section 2), we identified 247 NQO1‐regulated ASEs: 73 examples of intron retention (IntronR), 47 examples of exon skipping (ES), 21 cassette exons, 37 alternative 5′ splice sites (A5SS), two examples of alternative 5′ splice site and exon skipping (A5SS&ES), 39 alternative 3′ splice sites (A3SS), three examples of alternative 3′ splice site and exon skipping (A3SS&ES), 10 mutually exclusive 5′ UTRs (5pMXE), and eight mutually exclusive 3′ UTRs (3pMXE) (Figure 4a). These results suggest that NQO1 knockdown has an extensive influence on splicing. Figure 4b shows four genes (Eea1, Stag2, Pdgfa, and Stk11ip) whose expression and alternative splicing were regulated by NQO1 in PC12 cells.

To explore the potential functions of NQO1‐regulated alternative splicing of genes, we analyzed the genes for enrichment in GO terms. They were highly enriched in axonogenesis, metabolic processes, neuron migration, apoptotic processes, signal transduction, protein phosphorylation, and positive regulation of apoptotic processes (Figure 4c). Enriched KEGG pathways included biotin metabolism, endocytosis, cell cycle, and mitogen‐activated protein kinase (MAPK) signaling (Figure 4d). These results suggest that by regulating the alternative splicing of transcription factors and co‐activators, NQO1 knockdown regulates the expression of genes mainly related to apoptosis.

We validated the results of our transcriptomic analysis using RT‑qPCR (Figures 4e,f and 1). Two important ASEs were located in BIN1 and Picalm, which have been linked to AD pathology and belong to apoptotic pathways.

## 4 DISCUSSION

Neuronal apoptosis is one of the main manifestations of AD (Cavallucci & D'Amelio, 2011; Colurso et al., 2003; Obulesu & Lakshmi, 2014; Radi et al., 2014). In addition to its traditional oxidoreductase activity, NQO1 has been reported to directly regulate apoptosis as well (Liu et al., 2015). Here, we explored the RNA‐binding function of NQO1 in AD. Knockdown of NQO1 promoted PC12 cell apoptosis, suggesting that NQO1 is an apoptotic regulator in neural cells. We further found that NQO1 knockdown altered the expression of numerous genes, upregulating genes involved mostly in negative regulation of neuron apoptotic processes and aging, while downregulating genes involved mostly in nervous system development, response to oxidative stress, and other pathways related to AD.

In our study, NQO1 knockdown upregulated Cryab, NGF, and Apoe in PC12 cells, while it downregulated Stat3. All these genes have previously been linked to AD pathology. αB‐crystallin (CryaB) plays a key role in various cellular processes such as differentiation, apoptosis, and gene expression (Clark, 2016). CryaB can reduce cytotoxicity of Aβ by inhibiting lengthening of fibrils (Dehle et al., 2010), and it can prevent aggregation or misfolding of proteins, enabling their correct refolding (Bakthisaran et al., 2015). Phosphorylation of CryaB via a p38 MAPK‐dependent mechanism may participate in AD (Muraleva et al., 2019). Nerve growth factor (NGF) regulates differentiation, growth, survival, and plasticity of neurons in the central nervous system (Niewiadomska et al., 2011), and it is being explored as a potential AD therapy (Mitra et al., 2019). Apoe isoforms have been strongly linked to AD risk, and they appear to help control the transport of brain lipids, neuronal signaling, mitochondrial function, glucose metabolism, and neuroinflammation (Uddin et al., 2019). Apoe4‐targeted therapeutic strategies show promise (Belloy et al., 2019). Phosphorylated STAT3 is linked to suppression of the transcription factor NF‐κB and caspase‐3, which may help protect against AD‐like pathology (Marrero et al., 2010). Stat3‐mediated astrogliosis may be a useful therapeutic target in AD (Reichenbach et al., 2019). In light of these previous findings, our results suggest that NQO1 may help protect against AD by maintaining apoptosis in the brain under control. Our findings highlight the need for more extensive studies into the RNA‐binding functions of NQO1.

Indeed, we found that NQO1 knockdown influenced not only the expression of numerous genes, but also their alternative splicing. According to bioinformatics analysis and RNA‐seq data, NQO1 also regulates the alternative splicing of many genes that were enriched positive regulation of apoptotic processes and MAPK signaling. Among the five distinct functional modules of the MAPK family, p38 MAPK appears to be the most essential regulator of AD pathology (Kheiri et al., 2018; Munoz & Ammit, 2010). The apoptosis signal‐regulating kinase 1, involved in pathologic apoptosis in neurodegenerative disorders (Kim & Choi, 2010), is an MAPK kinase that enables p38 MAPK phosphorylation and acts via caspase 3 to initiate apoptosis dependent on amyloid precursor protein (Lauretti & Praticò, 2015; Liao et al., 2004). Inhibition of p38 MAPK hinders Aβ‐induced apoptosis in PC12 cells (Song et al., 2004). While p38 MAPK inhibitors have shown potential to prevent progression of Aβ and tau pathology in AD, they can cause side effects and can cross‐react with other kinases, limiting their clinical usefulness (Kheiri et al., 2018). Fruitful leads for therapeutic development may come from studies that explore how NQO1 acts as an RBP to influence p38 MAPK pathways.

We found evidence that NQO1 regulates splicing of the genes encoding phosphatidylinositol‐binding clathrin assembly protein (PICALM) and bridging integrator 1 (BIN1). Both genes have been identified as AD risk factors in genome‐wide association studies (Harold et al., 2009; Van Cauwenberghe et al., 2016). PICALM and BIN1 may influence processing of amyloid precursor protein, altering Aβ levels and thereby affecting AD risk (Harold et al., 2009; Tan et al., 2013). BIN1 has also been connected to tau pathology, but the pathways involved are unclear (Tan et al., 2013). BIN1 plays a role in cell cycle control and can mediate cell apoptosis via a caspase‐independent process (Elliott et al., 2000; Galderisi et al., 1999) but mechanistic details are unclear. Also unclear is whether BIN1‐related apoptosis is involved in AD (Gao et al., 2021). Future studies should explore these two proteins in AD in greater detail, together with NQO1.

Our study presents several limitations: first, our study currently involved in vitro experiments, and the PC12 cell line has many defects as an AD cell model. The results of this study need to be further verified in AD animal models and AD patient biological specimens. In addition, presently our study does not explain specifically how NQO1 regulates these genes and which role NQO1 plays in regulating these genes in the pathophysiology of AD.

Despite these limitations, our genome‐wide transcriptional and splicing analyses of NQO1 knockdown cells and control cells provide strong evidence that NQO1 posttranscriptionally regulates many genes involved in apoptosis. This regulation may make NQO1 an important player in the complex pathways leading to AD.

## AUTHOR CONTRIBUTIONS

Yingshi Du and Yongming Liu conceived the project and designed and supervised the experiments. Dong Chen, Jing Wang, and Yue Song performed the experiment. Gejing Liu, Qian Zhang, and Jinggang Yang analyzed the data. Yingshi Du drafted the manuscript. All authors reviewed the draft manuscript and approved the final version of the manuscript.

## CONFLICT OF INTEREST STATEMENT

The authors declare no conflicts of interest.

### PEER REVIEW

The peer review history for this article is available at https://publons.com/publon/10.1002/brb3.2917.

## Supporting information

Supplementary Figure 1. NQO1‐regulated cassette exon of BIN1 and verification by RT‐qPCR.

Supplementary Table 1. Primers sets related to the experimental procedures

## Data Availability

RNA‐seq data in this publication have been deposited in NCBI's Gene Expression Omnibus and are accessible through GEO series accession number. All additional data analyzed during this study are included in this published article and its additional files.
