# Supplementary figures and images for: NQO1 regulates expression and alternative splicing of apoptotic genes associated with Alzheimer's disease in PC12 cells
Source: Brain Behav. 2023 Mar 31;13(5):e2917. doi: 10.1002/brb3.2917 (PMC10175992; doi:10.1002/brb3.2917)

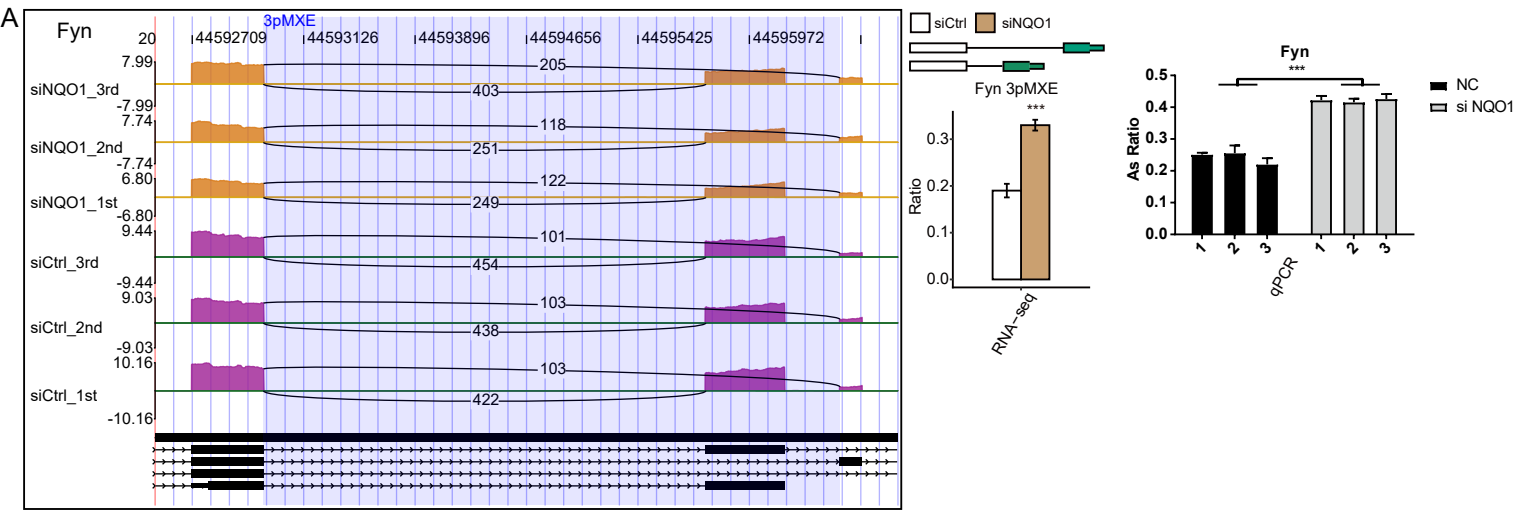

Supplement: Supplementary file 1 — Supplementary Figure 1. NQO1‐regulated cassette exon of BIN1 and verification by RT‐qPCR. [file BRB3-13-e2917-s002.pdf]
