# Supplementary material for: NQO1 regulates expression and alternative splicing of apoptotic genes associated with Alzheimer's disease in PC12 cells
Source: Brain Behav. 2023 Mar 31;13(5):e2917. doi: 10.1002/brb3.2917 (PMC10175992; doi:10.1002/brb3.2917)
Supplement: Supplementary file 2 — Supplementary Table 1. Primers sets related to the experimental procedures [file BRB3-13-e2917-s001.docx]

Supplementary Table 1. Primers sets related to the experimental procedures

| Gene | Primer | Sequence (5'-3') | Related Figures |
| --- | --- | --- | --- |
| GAPDH(Rat) | Forward | TCTCTTGTGACAAAGTGGACA | Figure 1A |
|  | Reverse | CCCATTCTCAGCCTTGACTGT |  |
| NQO1 | Forward | CTACACGTATGCCACCATGT | Figure 1A |
|  | Reverse | ATTGGCCAGAGAATGACGTT |  |
| Lgmn | Forward | CTGTGAAGGCGACAAGAG | Figure 3 |
|  | Reverse | GGTCTGGAGGAAGGAACT |  |
| Cryab | Forward | TCCTCTCACCATTACTTCTTC |  |
|  | Reverse | CTAGTCACAGATGTTCATTCG |  |
| Brd7 | Forward | TGAACAAGTGACCAATAACC |  |
|  | Reverse | AATCTACGAGGCTGTGTC |  |
| Ngf | Forward | TCTTCGGACACTCTGGATT |  |
|  | Reverse | CGTGGCTGTGGTCTTATC |  |
| Apoe | Forward | AGGCAATGGAGTTGGTAG |  |
|  | Reverse | CAAGATGGAGGAGCAGAC |  |
| Stat3 | Forward | AGGATTGGAAGCCATCAAC |  |
|  | Reverse | GCTGATCTGTCTGTGGAAT |  |
| Picalm | Model forward | ATGTTTGTTGGATTCAGTCC | Figure 4 |
|  | AS forward | ACATGGGGAGGATTCAGTCC |  |
|  | Model/AS reverse | ACCAGAATCTACAGCAAC |  |
| Bin1 | Model forward | CCCATCTCAGAGCTCTCTCC |  |
|  | AS forward | AAGCGACCTCCAGCTCTCTCC |  |
|  | Model/AS reverse | AAGCGTCCAGTCGTAGTGC |  |
| Fyn | Model forward | ACTACATCACCACGAGGGC | Figure S1 |
|  | AS forward | TCAGCTTTCTCTGAGTAATG |  |
|  | Model/AS reverse | GCGGCTCTCTCTGAGTAATG |  |
